# Growing a peritoneal dialysis program in Palestine from zero to 178 patients in 5 years: a single-center experience

**DOI:** 10.1186/s12882-023-03064-x

**Published:** 2023-01-26

**Authors:** Ameed Taher, Ahmad Enaya, Ala Sarsour, Sami Bahar, Dalia Hamayel, Omar Safarini, Zakaria Hamdan, Zaher Nazzal

**Affiliations:** 1 Department of Medicine, Faculty of Medicine and Health Sciences, An-Najah National University, Nablus, Palestine; 2 Department of Internal Medicine, An-Najah National University Hospital, Nablus, Palestine; 3 Kidney and Dialysis Section, An-Najah National University Hospital, Nablus, Palestine

**Keywords:** Experience, Peritoneal Dialysis, PD discontinuation, Peritonitis, Mortality

## Abstract

**Introduction:**

Kidney failure is rapidly rising in Palestine, as the number of patients receiving maintenance dialysis has quadrupled in the last 15 years. In this study, we share an overview of our experience growing a peritoneal dialysis (PD) program from zero to 178 patients in 5 years at An-Najah National University Hospital in Palestine, presenting some challenges and ways to overcome them.

**Methods:**

This was a single-center retrospective study of patients treated with PD from November 2016 to December 2021. Demographic and clinical data were obtained for each patient. In addition, PD discontinuation, peritonitis, and mortality rates were calculated and presented as the primary patient outcomes.

**Results:**

A total of 158 patients were eligible for the study. The mean age was 51.8 ± 16.4 years, and 53.8% of patients were male. Diabetic nephropathy was the most common cause of kidney failure. 63 episodes of peritonitis were diagnosed in 48 patients (30.4%) for a rate of 1 episode/ 38.2 patient-months (0.31 episodes/ patient-years). 20 patients had their PD treatment discontinued, mainly due to psychosocial reasons and infectious and mechanical complications. Death was the fate of 27 patients, with cardiovascular disease and COVID-19 being the two main causes.

**Conclusion:**

The outcomes of this experience proved favorable and showed that PD could serve as a viable option for kidney failure patients in Palestine. Moreover, this study can serve as an example for other places where circumstances are challenging to take the initiative of starting their PD programs.

## Introduction

Kidney failure is a rapidly increasing global health and healthcare burden [1]. Patients with kidney failure need Kidney Replacement Therapy (KRT) for survival. KRT modalities include kidney transplantation, hemodialysis (HD), and peritoneal dialysis (PD). Although kidney transplantation is the preferred treatment for eligible kidney failure patients, dialysis remains the predominant therapy worldwide [1]. The vast majority of the global dialysis population receives HD, while PD patients represent just about 11% of the mentioned population [2]. PD remains underutilized even though many studies have shown that it offers kidney failure patients a better quality of life while at the same time producing similar survival outcomes when compared to HD [3, 4]. This underutilization can be explained by inadequate awareness of PD among physicians and patients, a dearth of trained medical personnel, and a lack of advocacy for it as a viable KRT modality [5, 6]. Nevertheless, leading global kidney disease organizations have been increasingly advocating for increased access and uptake of PD [7], especially after the COVID-19 pandemic hit, as many studies have demonstrated lower infection rates among PD patients compared to their HD counterparts [8, 9].

While the incidence of kidney failure has stabilized or even decreased in some developed countries [10], it has become a significantly growing problem in the developing world [11], and Palestine is not an exception. In 2006, the total number of patients on dialysis in the West Bank was 391 [12], while in 2021, it stood at 1554 [13], a whopping four-fold increase in a matter of 15 years only. Since both the life expectancy and the incidence of diabetes and hypertension are projected to rise even more in the coming years, so will the number of Palestinian kidney failure patients. Moreover, as in other developing countries, kidney transplantation services are limited in Palestine, so this dramatic rise in kidney failure patients will be translated into a significantly bigger dialysis population.

**Fig. 1:**
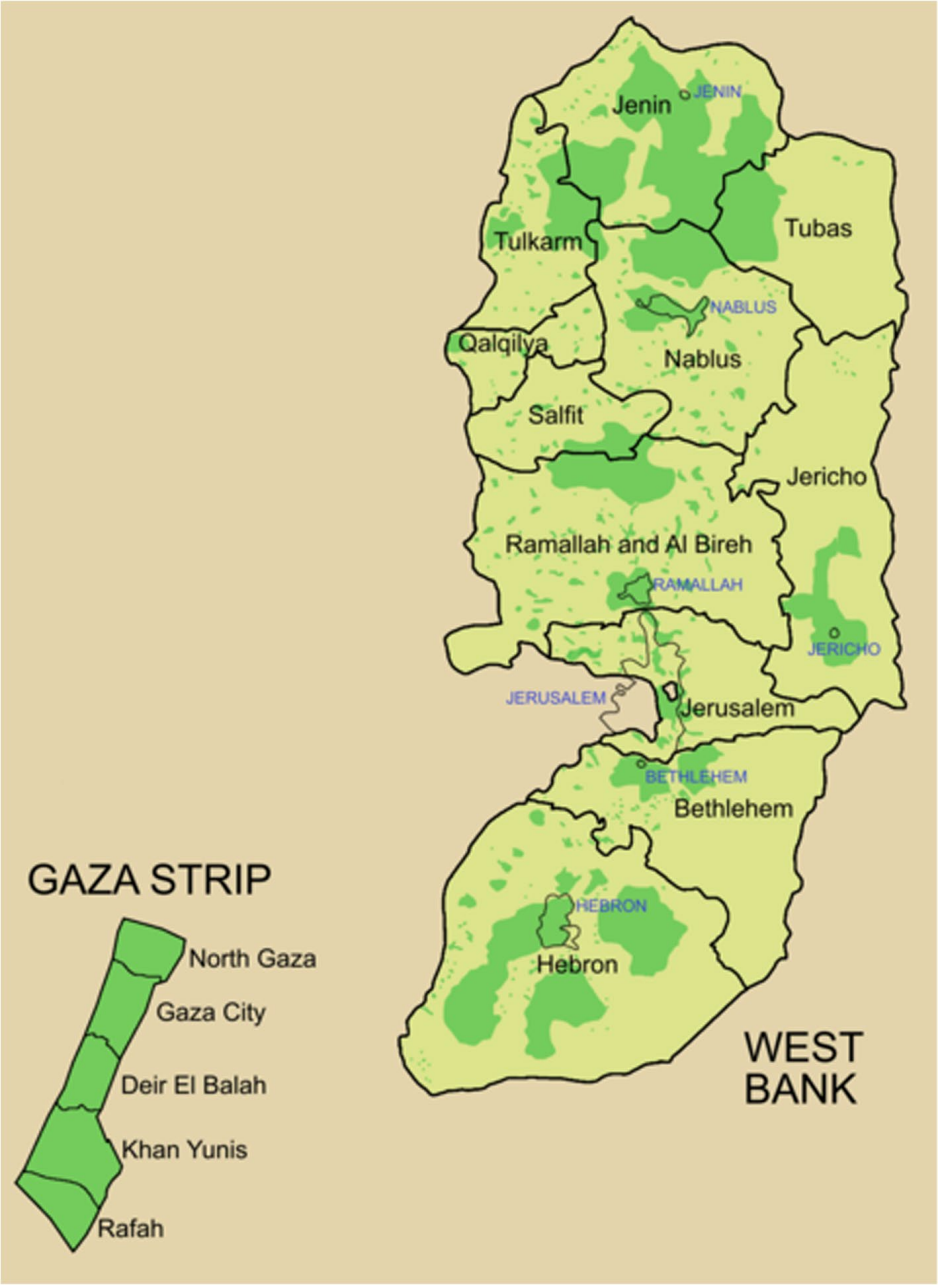
Governorates of the State of Palestine. Green indicates Palestinian enclaves within the West Bank, in addition to Gaza Strip, which is completely cut off from the West Bank. Yellow areas are under direct Israeli control. credit line: © Roke~commonswiki / Wikimedia Commons / CC-BY-SA-3.0

The State of Palestine is a lower-middle income country located in Western Asia, at the southeast corner of the Mediterranean Sea. It comprises three separate entities: The West Bank (WB), East Jerusalem, and Gaza Strip, all of which are under occupation. As a result, movement between these three entities is extremely limited. Furthermore, the WB itself (with an area of over 5600 km2 and a population of around 3 million people) is composed of numerous Palestinian enclaves, movement among which is also heavily controlled by Israel. It also has many Israeli settlements between the Palestinian enclaves, and violence in these areas is frequent (Fig. 1).

This fragmentation policy has been shown to have a devastating impact on the economy, social networks, and the provision of essential services such as healthcare [14]. HD patients are an especially vulnerable group in this respect, as they need to regularly attend one of the 11 dialysis centers scattered across the WB but are always under the threat of facing checkpoints, curfews, and settler violence on their way to treatment, making their treatment journeys long, challenging, and potentially unsafe. This, in addition to the quality-of-life advantages PD offers, the possibility of HD failure in some patients, and the overcrowding that we had experienced in our HD center due to a rising incidence of kidney failure, prompted us to establish a PD program in the WB in 2016. Furthermore, PD eliminates the need for patient transportation to HD centers, making it an attractive alternative to HD in areas experiencing conflicts and disasters [15]. In this study, we share our experience growing a PD program from zero to 178 patients in 5 years, presenting some challenges and ways to overcome them. Specifically, we aimed to assess PD discontinuation, peritonitis, and mortality rates as the main patient outcomes.

## Methodology

This was a single-center retrospective study of patients treated with PD from November 2016 to December 2021 at An-Najah National University Hospital in Nablus. All kidney failure patients who were at least 18 years of age and who had their PD treatment initiated were included. All patients underwent a double cuff Trenchoff catheter implantation and received continuous ambulatory peritoneal dialysis (CAPD) with a Y-set connection system for exchanges (Fresenius Medical Care, Bad Homburg, Germany). Catheter implantation was performed surgically by one surgeon at the beginning of the study period. Then the number grew to three surgeons by the end, in addition to a few patients who underwent percutaneous implantation. Patients had four exchanges per day in general and used conventional PD solutions (containing 1.5, 2.5, and 4.25% dextrose). All patients received training by one of two nurses at the initiation of their treatment, which lasted for an average of 10 days at the beginning of the study period, then gradually decreased to 5 days by the end. They were also given contact numbers for the PD nurses to respond to their concerns and/or inquiries if any arose. Continuous communication with patients through WhatsApp was maintained and helped us intervene prior to the development of major problems. Patients were encouraged to make once-a-month visits as well. The Palestinian government bears all the costs of kidney failure treatments, so no patient endured any out-of-pocket payments. Medications commonly prescribed for PD patients are readily available in Palestine, and are also paid for by the government. In addition, they are responsible for combating chronic diseases such as diabetes mellitus and hypertension, as well as securing medications such as anti-hypertensives or anti-diabetic drugs, as well as nephroprotective drugs such as Renin-angiotensin-aldosterone system inhibitors, at very low costs.

Demographic and clinical data were obtained for each patient, which included age, gender, educational level, place of residence, body mass index, smoking status, the reason behind PD choice, etiology of kidney failure, and the presence of comorbidities. In addition, all infectious and non-infectious complications were recorded as well as treatment outcomes. Outcome measures included patient mortality (defined as death due to any cause), PD discontinuation (defined as patient switching from PD to HD for any reason for at least 30 days), and rate of peritoneal dialysis-associated peritonitis (diagnosed when at least two of the following three criteria are present: 1) the presence of abdominal pain and/or cloudy dialysis effluent, 2) a dialysis effluent white cell count above 100/μL or with over 50% of polymorphonuclear leukocyte in the differential count, 3) a positive dialysis effluent culture).

The data were analyzed to determine the incidence and the associated factors of peritonitis, PD discontinuation, and mortality. We utilized Microsoft Office Excel and version 26 of Statistical Package for the Social Sciences (SPSS) for data entry and analysis. Categorical variables were displayed as frequency and percentages, whereas continuous variables were presented as mean and standard deviation (SD). We used the strategy of omitting missing data and analyzing the remaining data to deal with missing data because the number of missing data in this study was minimal. We employed the Chi-square test and independent t-test, as appropriate, to evaluate risk factors of PD outcome measures; peritonitis, PD discontinuation, and mortality. We used the multivariable regression model to control for confounders and assess predictors of peritonitis, PD discontinuation, and mortality. The significance level was set at .05. The study was approved by the An-Najah National University Institutional Review Board committee [Reference #: Med. Feb. 2021/15], and the research team ensured that all patient data remained confidential.

## Results

### Background and clinical characteristics

Over 5 years, 178 kidney failure patients underwent PD in our dialysis unit. Out of these, only 158 patients were included in this study, as 20 patients were under the age of 18 and were, therefore, excluded (Table 1). Eighty-five patients (53.8%) were male, 74 (46.8%) came from outside the Nablus Governorate (where the dialysis unit is located), and 86 (54.4%) received no more than primary education (below tenth grade). The study patients’ mean age was 51.8 ± 16.4 years, ranging from 18 to 89 years, and their mean BMI was 25.4 ± 5.8, ranging from 16.3 to 50.8. Hypertension (77.8%), diabetes (47.5%), and heart disease (36.1%) were the most common comorbidities reported among patients. Their kidney failure was caused primarily by diabetic nephropathy (42.4%), glomerular disease (10.8%), and hypertensive nephropathy (8.9%). Only 17.1% had PD as their first treatment modality, with vascular access failure being the most common reason for the change of modality to PD (47.5%). The average length of dialysis was 15.3 ± 13.9 months, ranging from 3 days to 63.1 months, and 55 (34.8%) patients required caregiver assistance (family member) to perform PD.

**Table 1:** Study participants’ demographic characteristics and comorbidities (*n* = 158)

	Frequency (%)	Mean ± SD
**Age** in years		51.8 ± 16.4
**Gender**		
Male	85 (53.8%)	
Female	73 (46.2%)	
**Educational level**		
Primary education or below	86 (54.4%)	
Secondary education or above	72 (45.6%)	
**Residence**		
Nablus	84 (53.2%)	
Others (outside Nablus)	74 (46.8%)	
**BMI**		25.4 ± 5.8
**Smoking** (yes)	62 (39.2%)	
**Comorbidities**		
Diabetes	75 (47.5%)	
Hypertension	123 (77.8%)	
Heart Disease	57 (36.1%))	
Liver Disease	11(7.0%)	
Respiratory Disease	5 (3.2%)	
**Causes of kidney failure**		
Diabetic nephropathy	67 (42.4%)	
Glomerular diseases	17 (10.8%)	
Hypertensive nephropathy	14 (8.9%)	
Nephrolithiasis	5 (3.2%)	
Others	19 (12.0%%)	
Unknown	36 (22.8%)	
**Duration of dialysis (**months)		15.3 ± 13.9
**Reasons for PD**		
Vascular access failure	75 (47.5%)	
Doctor preference	55 (34.8%)	
Patient preference	28 (17.7%)	
**Catheter insertion technique**		
Surgical	150 (94.9%)	
Percutaneous	8 (5.1%)	
**Residual Urine** (yes)	41 (25.9%)	
**PD was the first modality of treatment** (yes)	27 (17.1%)	
**Need for Caregiver** (yes)	55 (34.8%)	

### PD complications and outcomes

Complications and outcomes are presented in Table 2. 63 episodes of peritonitis were diagnosed in 48 patients (30.4%) for a rate of 1 episode/ 38.2 patient-months (0.31 episodes/ patient-years), while 110 patients (69.6%) had no peritonitis episodes during the study period. One patient experienced four episodes, and four patients had three episodes each. Furthermore, microbiological cultures were negative in only seven episodes (11.1%). As for the other complications, only seven patients (4.4%) had an exit-site infection, while 41 mechanical complication episodes were experienced by 29 patients (18.4%), with leakage being the most common mechanical complication encountered.

**Table 2:** Frequency and distribution of PD complications and outcomes (n = 158)

	Frequency (%)
**Peritonitis** (yes)	48 (30.4%)
**Exit site infection** (yes)	7 (4.4%)
**Mechanical complications (any)**	29 (18.4%)
Leakage	11 (7.0%)
Hernia	8 (5.1%)
Catheter migration or malposition	9 (5.7%)
Adhesions	8 (5.1%)
Hematoma	5 (3.2%)
**Outcome**	
Active PD	109 (69.0%)
PD discontinuation	20 (12.7%)
Kidney transplant	2 (1.2%)
Death	27 (17.1%)
**Reasons for PD discontinuation** (*n* = 20)	
Psychosocial	7
Peritonitis	6
Mechanical complications	5
Ultrafiltration failure	1
Pancreatitis	1
**Causes of mortality** (*n* = 27)	
Cardiovascular diseases	9
COVID-19	6
Calciphylaxis	3
Peritonitis	2
Other infections	3
Others	4

A total of 109 patients (69.0%) were still active on PD at the end of the study, while 27 (17.1%) had died, 20 (12.7%) had experienced PD discontinuation, and only 2 (1.2%) had undergone kidney transplantation. PD discontinuation was due to psychosocial factors in 7 cases, peritonitis in 6 patients, and mechanical complications (leakage and adhesions) in 5 cases. The two leading causes of death were cardiovascular disease (9 patients) and Covid-19 (6 patients), while peritonitis was the culprit in two patients.

### Factors associated with peritonitis, PD discontinuation, and mortality

Tables three, four, and five present the study findings on the risk factors associated with peritonitis, PD discontinuation, and mortality. The risk of peritonitis was higher among patients residing outside Nablus [*p*-value = .045; aOR = 2.6 (95% CI: 1.1–5.0)], patients who had no residual urine at the time of PD initiation [p-value .012; aOR = 6.2 (95% CI: 1.5–25.3)], and those who had experienced mechanical complications [p-value .002; aOR = 4.8 (95% CI: 1.8–12.8)] (Table 3).

**Table 3:** Background and clinical characteristics of peritoneal dialysis patients with peritonitis

	Peritonitis			Multivariable Analysis	
	Yes	No	***P***- value*	aOR (95%CI)	a***P***- value
**Age** in years	50.3 ± 16.7	52.4 ± 16.2	.458	1.1 (.97–1.2)	.819
**Gender**					
Male	24 (28.2%)	61 (71.8%)	.565	1	
Female	24 (32.9%)	49 (67.1%)		1.1 (.46–2.5)	.893
**Body Mass Index**	26.6 ± 5.9	24.8 ± 5.7	.082	1.1 (.98–1.2)	.120
**Educational level**					
Primary education or below	27 (31.4%)	59 (68.6%)	.798	1.6 (.64–4.1)	.307
Secondary education or above	21 (29.2%)	51 (70.8%)		1	
**Residence**					
Nablus	19 (22.6%)	65 (77.4%)	.021	1	
Others (outside Nablus)	29 (39.2%)	45 (60.8%)		2.6 (1.1–5.0)	.045
**Smoking**					
Yes	19 (30.6%)	43 (69.4%)	.920	1.3 (.52–3.2)	.576
No	29 (30.2%)	67 (69.8%)		1	
**Diabetes Mellitus**					
Yes	24 (32.0%)	51 (68.0%)	.638	1.5 (.68–3.8)	.407
No	24 (28.9%)	59 (71.1%)		1	
**Residual Urine**					
Yes	6 (14.6%)	35 (85.4%)	.011	1	
No	42 (35.9%)	75 (64.1%)		6.2 (1.5–25.3)	.012
**PD was the first modality**					
Yes	7 (25.9%)	20 (74.1%)	.596	1	
No	41 (31.3%)	90 (68.7%)		1.6 (.39–6.8)	.498
**Need for Caregiver**					
Yes	16 (29.1%)	39 (70.9%)	.826	1.3 (.50–3.2)	.624
No	32 (31.1%)	71 (68.9%)		1	
**Any mechanical complication**					
Yes	18 (48.6%)	19 (51.4%)	.005	4.8 (1.8–12.1)	.002
No	30 (24.6%)	92 (75.4%)		1	

*Chi-squared test and independent t-test, **aOR:** adjusted odds ratio, **CI:** Confidence interval, **a*P*- value:** adjusted *P*- value

The need for a caregiver to assist in PD performance was associated with a higher risk of PD discontinuation [p-value .001; aOR = 1.6 (95% CI: 1.3–8.6)] (Table 4), while the two factors found to be significantly associated with an increased risk of mortality were not having residual urine at the time of PD initiation [p-value .006; aOR = 19.6 (95% CI: 2.6–32.2)] and not having PD as the first modality of treatment [p-value .033; aOR = 6.4 (95% CI: 1.2–26.7)] (Table 5).

**Table 4:** Background and clinical characteristics of peritoneal dialysis patients with PD discontinuation

	PD discontinuation			Multivariable Analysis	
	Yes	No	***P***- value*	aOR (95%CI)	a***P***- value
**Age** in years	59.4 ± 16.9	50.7 ± 16.1	.027	1.1 (.97–1.2)	.470
**Gender**					
Male	13 (15.3%)	72 (84.7%)	.405	1.1 (.37–2.8)	.990
Female	7 (9.6%)	66 (90.4%)		1	
**Body Mass Index**	26.9 ± 5.4	25.1 ± 5.9	.195	.92 (.83–1.08)	.068
**Educational level**					
Primary education or below	16 (18.6%)	70 (81.4%)	.014	1.6 (.54–4.5)	.412
Secondary education or above	4 (5.6%)	68 (94.4%)		1	
**Residence**					
Nablus	13 (15.5%)	71 (84.5%)	.193	1.9 (.75–4.9)	.150
Others (outside Nablus)	7 (9.5%)	67 (90.5%)		1	
**Smoking**					
Yes	9 (14.5%)	53 (85.5%)	.697	1.3 (.46–3.8)	.605
No	11 (11.5%)	85 (88.5%)		1	
**Diabetes Mellitus**					
Yes	15 (20.0%)	60 (80.0%)	.017	1.3 (.45–3.8)	.615
No	5 (6.0%)	78 (94.0%)		1	
**Residual Urine**					NA^**†**^
Yes	0 (0.0%)	41 (100.0%)	.004	1	
No	20 (13.2%)	97 (82.9%)			
**PD was the first modality**					NA^**†**^
Yes	0 (0.0%)	27 (100.0%)	.030	1	
No	20 (15.3%)	111 (84.7%)			
**Need for Caregiver**					
Yes	13 (23.6%)	42(76.4%)	.002	1.6 (1.3–8.6)	.001
No	7 (6.8%)	96 (93.2%)		1	
**Any mechanical complication**					
Yes	6 (20.7%)	23 (79.3%)	.150	2.0 (.61–7.8)	.322
No	14 (10.9%)	115 (89.1%)		1	
**Peritonitis**					
Yes	9 (18.8%)	39 (81.2%)	.128	1.5 (.59–4.1)	.368
No	11 (10.0%)	99 (90.0%)		1	

*Chi-squared test and independent t-test, **aOR:** adjusted odds ratio, **CI:** Confidence interval, **a*P*- value:** adjusted P- value. ^**†**^Variables with zero frequencies were removed from the multivariable model

**Table 5:** Background and clinical characteristics of peritoneal dialysis patients with mortality

	Mortality			Multivariable Analysis	
	Yes	No	***P***- value*	aOR (95%CI)	a***P***- value
**Age** in years	55.5 ± 18.8	50.9 ± 15.8	.189	1.1 (.97–1.2)	.466
**Gender**					
Male	14 (16.5%)	71 (83.5%)	.824	1	
Female	13 (17.8%)	60 (82.2%		1.8 (.60–5.6)	.296
**Body Mass Index**	23.9 ± 4.9	25.6 ± 6.0	.164	.89 (.80–1.2)	.114
**Educational level**					
Primary education or below	18 (20.9%)	68 (79.1%)	.161	.79 (.26–2.4)	.680
Secondary education or above	9 (12.5%)	63 (87.5%)		1	
**Residence**					
Nablus	12 (14.3%)	72 (85.7%)	.319	1	
Others (outside Nablus)	15 (20.3%)	59 (79.7%)		2.7 (.87–8.6)	.084
**Smoking**					
Yes	9 (14.5%)	53 (85.5%)	.490	.44 (.14–1.4)	.186
No	18 (18.8%)	78 (81.3%)		1	
**Diabetes Mellitus**					
Yes	15 (20.0%)	60 (80.0%)	.355	1.6 (.52–5.2)	.407
No	12 (14.5%)	71 (85.5%)		1	
**Residual Urine**					
Yes	2 (4.9%)	39 (95.1%)	.016	1	
No	25 (21.4%)	92 (78.6%)		19.6 (2.6–32.2)	.006
**PD was the first modality**					
Yes	5 (18.5%)	22 (81.5%)	.828	1	
No	22 (16.8%)	109 (83.2%)		6.4 (1.2–26.7)	.033
**Need for Caregiver**					
Yes	15 (27.3%)	40 (72.7%)	.013	1.5 (.63–5.0)	.250
No	12 (11.7%)	91 (88.3%)		1	
**Any mechanical complication**					
Yes	3 (10.3%)	26 (89.7%)	.286	1.8 (.41–7.9)	.436
No	24 (18.6%)	105 (81.4%)		1	
**Peritonitis**					
Yes	9 (18.8%)	39 (81.2%)	.714	.99 (.34–2.9)	.899
No	18 (16.4%)	92 (83.6%)		1	

*Chi-squared test and independent t-test, **aOR:** adjusted odds ratio, **CI:** Confidence interval, **a*P*- value:** adjusted *P*- value

## Discussion

Peritoneal dialysis was first introduced in Palestine in 1992 when Israel administrated the Palestinian healthcare system. It then changed hands to the newly established Palestinian Ministry of Health in 1994 after signing the Oslo Peace Accords. However, it lasted only a short time, as it ended in 1996, due to resource inadequacy (consumables not readily available), lack of financial support, and staff-training unavailability. The total number of patients who had received PD at the time was 20.

Twenty years later, two nephrologists with a strong interest in PD started over the program with several steps being made to revamp it. First, they began by qualifying nurses locally and employing part-time well-experienced nurses to help master the PD service. Second, an agreement was reached with a motivated surgeon to provide timely PD catheter placement service. Third, a KRT education program was organized by a dedicated nurse, which mandated education about all replacement modalities, not only for patients followed up in the outpatient clinic but also for those who started maintenance dialysis in the inpatient setting and other interested patients with no contraindication to PD. The KRT education program was a multidisciplinary PD educatory program attended by a nephrologist, a nurse, and patients. It helped patients overcome their fears and concerns about the modality and made them recommend the service to other patients on HD therapy. That is why it was essential to start the program with satisfactory outcomes. It allowed those initial PD patients to become the best tool to promote PD as a viable treatment modality to others. We constantly encouraged potential PD patients to meet current PD patients while educating them about PD so that they could relate their actual experiences with them and help address any questions or concerns that these potential patients might have in mind. This has helped considerably in promoting and expanding our program, and all these steps have significantly lessened obstacles faced and made this experience sustainable since 2016.

There was initially a concern regarding starting a PD program in Palestine because of a perception of an insufficient level of awareness and education among the population when compared to more advanced countries, which might lead to negative results, as many studies have established a link between low education levels and worse outcomes among PD patients [16]. Indeed, over half of our patients (54.4%) had received no more than primary education. This led us to provide patients with lengthy training time at the beginning of the study period, but it gradually decreased throughout as this lengthiness was deemed unnecessary. Moreover, the overall outcomes turned out to be favorable: the rate of peritonitis stood at 0.31 episodes per patient-years (1 episode per 38.2 patient months), achieving the target of having no more than 0.4 episodes per patient-years set by the International Society of Peritoneal Dialysis (ISPD) in 2022 [17]. Furthermore, only 11% of the peritonitis episodes yielded a culture-negative result, achieving the ISPD target of having no more than 15% culture-negative episodes [17].

Additionally, the crude mortality rate was calculated to be 107 per 1000 patient-years, which compares favorably with what has been reported in developed countries, such as the United States and New Zealand [18, 19]. Furthermore, COVID-19 was identified as the cause of death in six out of the 27 patients (22.2%) who passed away, which suggests that the mortality rate could have been even more favorable compared to that in the US and New Zealand since those studies took place before the emergence of the pandemic. Besides, a previous study that examined the impact of Covid-19 on the dialysis population in Palestine found that HD patients were more than three times more likely to acquire Covid-19 compared to their PD counterparts (37% vs. 11.3%) [13], adding yet another advantage that PD can provide to kidney failure patients.

Distance from the PD center, absence of residual urine output at the time of PD initiation, and mechanical complications were determined to be the factors associated with an increased risk of peritonitis. Prior studies have demonstrated that patients who live far from their dialysis units tend to have worse outcomes [20, 21]. Our data only demonstrated an association between distance and the rate of peritonitis, as those residing inside Nablus had significantly fewer peritonitis episodes than those living outside of it. It should also be noted that although the distance between Nablus and the other cities may not appear long per global standards, it does constitute a barrier to patients due to the fragmented nature of the WB and the poor quality of its infrastructure, which presumably explains the discrepancy between the two groups. Training healthcare providers about the basic principles of PD to meet essential patients’ needs could help alleviate this discrepancy.

The need for a family member to assist in PD performance was the only factor found to be associated with an increased risk of PD discontinuation on multivariable analysis. This is different from what has been found in a French study, which showed lower rates of PD discontinuation among patients receiving assisted PD [22]. This suggests that putting more emphasis on training family members could decrease the drop-out rates among those who cannot perform PD independently. It’s worth noting that there is no home nursing assistance in Palestine, and so assisted PD is offered exclusively by family members. Unlike in France, where both options are available.

The absence of residual urine output at the time of PD initiation and not having PD as the first treatment modality were associated with an increased risk of PD discontinuation on bivariate analysis but could not be studied in the multivariable model due to zero frequencies. These findings are consistent with what has been found in previous studies [23, 24]. It is worth noting that only 17% of our patients underwent PD as their first KRT modality. This suggests that more can be done when it comes to advocating for PD and raising awareness about the benefits it can provide among physicians and patients, which could lead to increased uptake of PD as the first treatment modality, which in turn could increase the time on PD therapy.

Other studies have found other factors linked with an increased risk of PD discontinuation, such as old age, diabetes, peritonitis, and male sex [25, 26]. The outcome disparity between the two sexes has been observed in some studies but has not been explained. In our experience, men were more likely to drop out of PD, but the difference was not statistically significant. It is worth mentioning, however, that all patients who stopped PD for psychosocial reasons in our study were men. Psychosocial reasons were the top cause of PD discontinuation in our data, which is different from what has been found in Australia/New Zealand, Netherlands, and France, where psychosocial factors played a smaller role compared to infectious and mechanical complications [27–29]. In our experience, patients’ realization of the commitment needed to perform PD and lack of adequate family support were the main drivers of PD discontinuation, which could be traced to suboptimal patient and family education before initiating PD.

The two factors associated with an increased risk of death were lacking residual urine output at the time of PD initiation and not having PD as the first treatment modality. Other suggested mortality-increasing factors like old age and diabetes were not statistically significant in our data, perhaps due to the limited sample size. Data on the relationship between peritonitis and overall mortality are conflicting, with some studies showing a negative relationship (a “peritonitis paradox”) [30] and others showing a positive association [31] or no relationship at all, as in our case. Another paradox is the “obesity paradox,” where obesity provides a survival benefit to dialysis patients [32], but the difference was not statistically significant in our data.

One limitation of our study was the relatively small sample size to determine the risk factors for peritonitis, PD discontinuation, and mortality. Additionally, since our data were obtained retrospectively, we could not determine the amounts of patients’ residual urine output, which would have helped us better understand its relationship with adverse outcomes, especially since it was the only factor associated with peritonitis, PD discontinuation, and mortality. Furthermore, long-term outcomes of PD therapy cannot be determined from this study since it is merely a five-year experience. Future studies are needed to report on these long-term outcomes.

Nonetheless, this was the first PD study that came out of Palestine. Despite having relatively limited resources, the favorable outcomes of our experience show that PD is a viable option for kidney failure patients in Palestine. Teamwork is a crucial determinant of success: Having an enthusiastic surgeon for catheter placement, a nephrologist willing to invest the time in establishing and maintaining the program, a dedicated nursing staff, and a supportive hospital administration made our program sustainable and successful outcomes feasible. We hope this study can serve as an example for other places where circumstances are challenging to take the initiative of starting their own PD programs.

## Data Availability

All data generated or analyzed during this study are included in this published article.
